# Regulation of survival in adult hippocampal and glioblastoma stem cell lineages by the homeodomain-only protein HOP

**DOI:** 10.1186/1749-8104-3-13

**Published:** 2008-05-28

**Authors:** Arianna De Toni, Marie Zbinden, Jonathan A Epstein, Ariel Ruiz i Altaba, Alain Prochiantz, Isabelle Caillé

**Affiliations:** 1 UMR CNRS 8542, ENS, rue d'Ulm, 75005 Paris, France; 2 University of Geneva Medical School, 8542 CMU, rue Michel Servet, CH-1211 Geneva 4, Switzerland; 3 Department of Cell and Developmental Biology, University of Pennsylvania School of Medicine, Curie Boulevard, Philadelphia, Pennsylvania 19104, USA; 4 Collège de France, place Marcelin Berthelot, 75231 Paris Cedex 05, France; 5 UFR de Biologie, Université Denis Diderot, 75013 Paris, France

## Abstract

**Background:**

Homeodomain proteins play critical roles in shaping the development of the embryonic central nervous system in mammals. After birth, neurogenic activities are relegated to stem cell niches, which include the subgranular layer of the dentate gyrus of the hippocampus. Here, we have analyzed the function of HOP (Homeodomain only protein) in this stem cell niche and in human glioblastomas.

**Results:**

We find that *HOP *is strongly expressed by radial astrocytes of the dentate gyrus in mice, which are stem cells that give rise to hippocampal granular neurons throughout adulthood. Deletion or down-regulation of *HOP *results in a decrease of apoptosis of these stem cells without changes in proliferation, and in an increase in the number of newly formed granule neurons. We also find that human glioblastomas largely lack *HOP *expression and that reintroduction of HOP function in glioma cells cultured as gliomaspheres leads to enhanced apoptosis in a subset of cases. In these cells, HOP function decreases clonogenicity.

**Conclusion:**

These data suggest that HOP participates in the regulation of the adult mouse hippocampal stem cell niche by negatively affecting cell survival. In addition, HOP may work as a tumor suppressor in a subset of glioblastomas. HOP function thus appears to be critical in the adult brain in a region of continued plasticity, and its deregulation may contribute to disease.

## Background

HOP (Homeodomain only protein; NM-175606) is a small 73 amino acid atypical homeodomain protein, composed simply of a homeodomain. HOP was first identified in the developing heart where it modulates cardiac growth [1,2]. Surprisingly, for a homeodomain protein, HOP cannot bind DNA but exerts its action by interacting with Serum responsive factor (SRF) and blocking its transcriptional activity. This interaction was proposed to regulate the balance between cardiomyocyte proliferation and differentiation. In addition, HOP was described as a tumor suppressor gene, as its expression is lost or low in lung cancer [3], head and neck squamous cell carcinoma [4], and choriocarcinoma, where its re-expression can inhibit cancer growth [5].

In the initial reports describing HOP in the heart, its expression was also detected in the developing neural tube and in the adult brain [1,2]. This, as well as its role as a regulator of differentiation in the heart, prompted us to assess a role for HOP in adult neurogenesis. Stem cell niches in the mouse forebrain's subventricular zone (SVZ) and the subgranular layer (SGL) of the dentate gyrus (DG) add new neurons to the olfactory bulb and the hippocampus, respectively, in a sustained manner. These processes are tightly regulated [6,7]. Cell death has been shown to be essential in the selection of newly formed neurons in the olfactory bulb [8,9] and DG [10,11]. However, even though apoptosis is an essential regulator of embryonic stem cell number [12,13] and despite the presence of apoptotic cells in both the SVZ and SGL [14], little is known about the regulation of apoptosis in adult stem cell niches.

Cell lineages in these niches have been described [15-17]. In the DG, the progenitors of new neurons are SGL radial astrocytes (called B or type 1 cells) [18,19]. These cells characteristically extend one or several radial processes across the entire granule cell layer, self-renew and give rise to immature intermediate precursors (D or type 2 cells), which divide and then mature into granule cells. Here, we show that *HOP *is expressed by radial astrocytic stem cells and increases neuronal production by promoting apoptosis of these cells.

It has been suggested that gliomas in general, and glioblastomas (GBMs) in particular, derive from the transformation of neural stem cells [20-22], which may give rise to cancer stem cells. These cells are rare tumor cells that self-renew, are tumorigenic and may be responsible for tumor maintenance, recurrence and possibly metastasis. Since cell death is crucially involved in the regulation of tumor formation and since normal brain stem cells and glioma stem cells share common regulatory mechanisms, we investigated a role for HOP in GBMs.

We show that *HOP *is down-regulated in GBMs. Its re-expression induces apoptosis in two of four GBM cultures tested and decreases GBM cancer stem cell clonogenicity in one of them. We conclude that HOP is a new regulator of stem cell survival in the adult brain and that its deregulation may participate in the tumorigenic process.

## Results

### SGL radial astrocytic stem cells in the hippocampus express HOP

**Figure 1:**
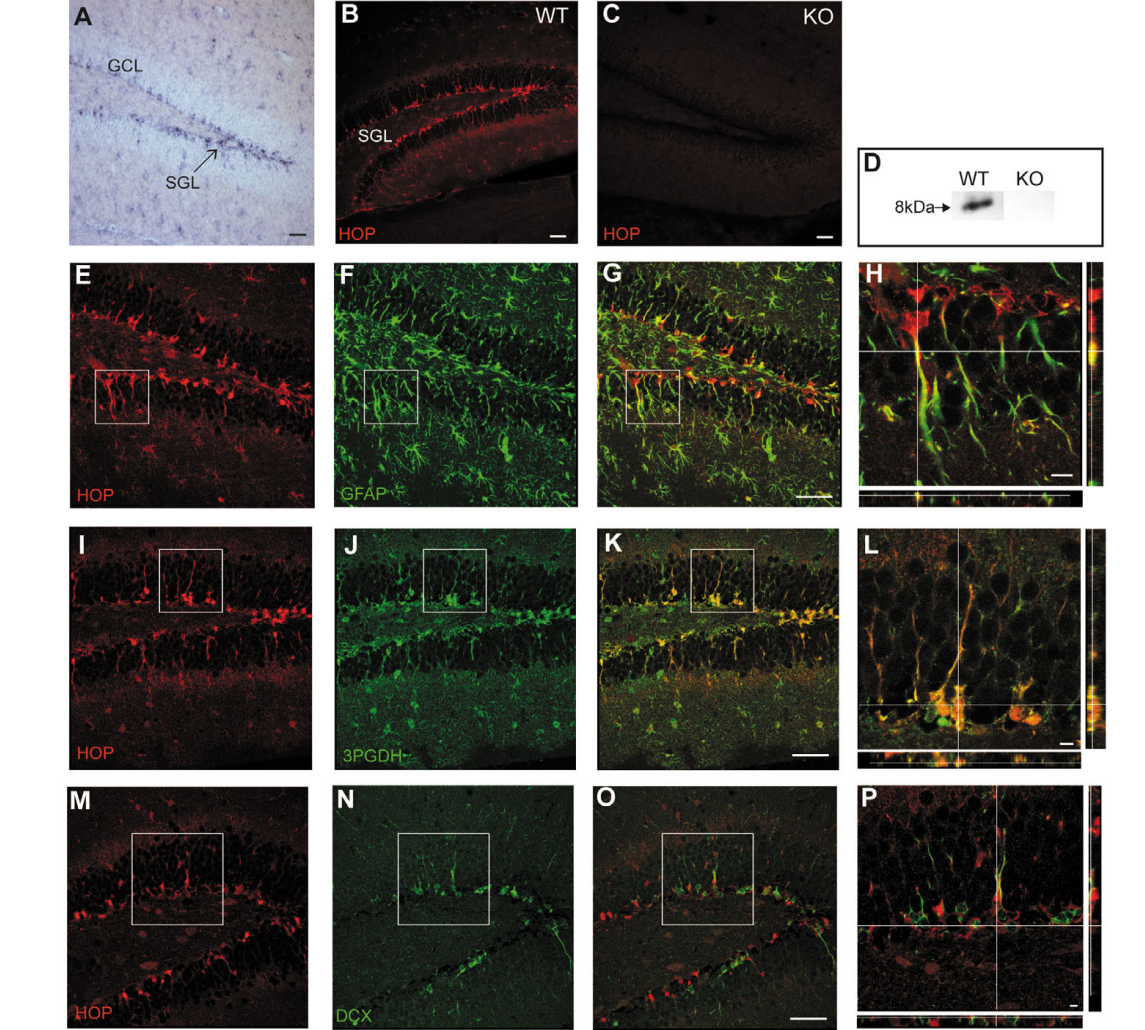
HOP is expressed by SGL radial astrocytes. **(a) ***In situ *hybridization shows *HOP *mRNA expression in numerous DG cells with a prominent labeling in the SGL (arrow), the neurogenic region for the production of granule cells destined to the granule cell layer (GCL). **(b) **Immunohistochemical detection of HOP protein in the DG, especially in the SGL. WT, wild type. **(c) **HOP immunohistochemical staining is absent in knock out (KO) mice, demonstrating HOP antibody specificity. **(d) **Western blot shows an 8 kDa protein from hippocampal wild-type extracts, which is absent in the knock out extracts. **(e-h) **Double-staining with HOP and GFAP antibodies, illustrating the co-expression of the two proteins. (h) Higher magnification of the boxed area in (e-g) with orthogonal projections along the z confocal stack at the x and y levels indicated by the intersecting white lines. **(i-l) **Double-staining with HOP and 3PGDH antibodies, showing that HOP immunoreactive cells are 3PGDH^+^. (l) Higher magnification of the boxed area with orthogonal projections. **(m-p) **Double-staining with HOP and DCX, showing that they are expressed by different SGL cells. (p) Higher magnification of the boxed area with orthogonal projections. The y projection shows, in particular, that DCX+ processes are closely juxtaposed to HOP+ processes but are distinct. Scale bars: (a-f, e-g, i-k, m-p), 50 μm; (h, l, p), 10 μm.

To localize *HOP *expression in detail in stem cell niches of the adult mouse brain, we performed *in situ *hybridization and immunolocalization studies. HOP mRNA and protein were not detected in the SVZ of the lateral ventricle of the adult forebrain. In contrast, they were found in the DG and, in particular, in cells of the SGL (Figure 1a,b). The specificity of the immunolabeling with the anti-HOP antibody that we raised was proven by the lack of staining in the SGL of the *HOP*^-/- ^mice [3] that survive the embryonic period (Figure 1c). Immunoblots of the hippocampus detected a single band at the expected size of 8 kDa, which was absent in *HOP*^-/- ^mice (Figure 1d).

The identity of HOP^+ ^cells was determined by double-labeling with glial fibrillary acidic protein (GFAP), a general marker of astrocytes (Figure 1e–h). HOP^+ ^cells were found to be GFAP^+ ^astrocytes, with SGL radial astrocytes displaying the highest levels. Use of a second marker, 3-phosphoglycerate dehydrogenase (3PGDH), which stains the cell bodies and processes of all astrocytes, including DG radial astrocytes [23], showed that all HOP^+ ^cells were 3PGDH^+^, thus confirming their astrocytic identity (Figure 1i–l).

SGL radial astrocytes give rise to immature neuronal precursors (D or type 2 cells), which are nested along astrocytic processes [16] and express doublecortin (DCX) [24,25]. Double immuno-staining with HOP and DCX showed that these cells do not express *HOP *(Figure 1m–p). Together, these results demonstrate that *HOP *is expressed by SGL radial astrocytes, which are neural stem cells in the adult hippocampus.

### HOP does not regulate the proliferation of SGL hippocampal stem cells

Given that adult neurogenesis requires proliferation and survival of stem cells and their progeny, we tested the role of HOP in these two processes.

**Figure 2:**
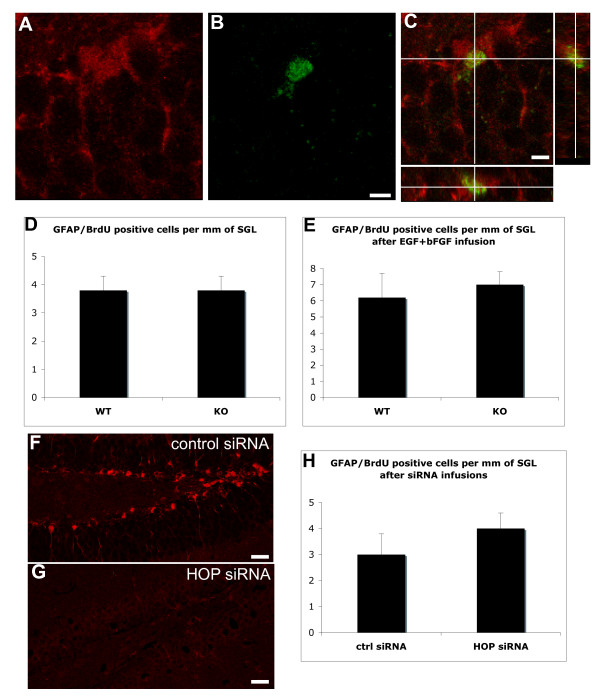
HOP does not regulate the proliferation of SGL astrocytic stem cells. **(a-c) **After a 2 h pulse of BrdU, mice were processed for HOP/BrdU immunocytochemistry. Some HOP immunoreactive cells incorporate BrdU. Scale bar: 10 μm. **(d, e) **After a 2 h pulse of BrdU, mice were processed for GFAP/BrdU ICC and the double-labeled SGL cells were counted under a confocal microscope (n = 5). The data are presented as the number of GFAP/BrdU cells per mm of SGL. The number of GFAP/BrdU cells is unchanged in the *HOP *knock out mice compared to the wild type (d) even when growth factors (EGF + bFGF, 20 μg/ml) were infused into the lateral ventricle to stimulate the proliferation of DG progenitors (n = 5) (e). **(f-h) ***HOP *(g) or control (f) siRNA coupled to the cell permeant peptide Penetratin (20 μM) were infused for three days above the DG to locally silence *HOP *expression. A strong reduction of *HOP *expression is seen after *HOP *siRNA infusion. (h) The number of GFAP/BrdU cells is not significantly different between the two conditions (n = 5). Scale bars: 50 μm.

HOP immunoreactive cells divide, as shown by short term (2 h) bromodeoxyuridine (BrdU) incorporation (Figure 2a–c), as described previously for SGL astrocytes [17,19,23]. We therefore tested whether HOP could regulate proliferation of SGL astrocytes by comparing the number of BrdU^+^/GFAP^+ ^in wild-type and *HOP*^-/- ^mice (n = 5). No significant differences were detected (Figure 2d). Moreover, no differences were observed between wild-type and *HOP*^-/- ^mice even after infusion of basic fibroblast growth factor (bFGF) and epidermal growth factor (EGF) to stimulate cell proliferation (n = 5; Figure 2e). This result was confirmed by comparing the number of 3PGDH^+^/BrdU^+ ^cells in wild-type and *HOP*^-/- ^mice (Additional file 1).

To confirm and extend this result, we knocked-down *HOP *in wild-type mice through RNA interference using cell-permeable small interfering RNAs (siRNAs). These siRNAs were rendered cell-permeable by reversibly coupling them to Penetratin, a small transducing peptide [26]. The disulfide bond linking the siRNA and Penetratin is broken in the intracellular context, allowing the siRNA to work in the nucleus [27]. Infusing *HOP *siRNA-Penetratin into the brain, at a location just above the DG with micro-osmotic pumps, led to a dramatic reduction in HOP immunoreactivity (Figure 2g) in the hippocampus, compared to the infusion of a control Penetratin-coupled siRNA (Figure 2f). Semi-quantification of the optical density revealed that HOP signal was reduced by 78% ± 5 standard error of the mean (SEM; n = 5). To avoid any possible non-specific effects due to direct injection damage of the DG, the infusions were repeated at a distance, into the lateral ventricle, and diffusion into the hippocampus was verified with a fluorescent siRNA. Lateral ventricle infusions of *HOP *siRNA resulted in a decrease of the optical density by 39% ± 3 SEM (n = 5), as compared with control siRNA infusions. *HOP*-siRNA and control siRNA-treated mice infused for three days in the lateral ventricle showed similar levels of BrdU incorporation in SGL GFAP^+ ^stem cells (Figure 2h). We conclude that HOP does not control the proliferation of hippocampal stem cells.

### Hippocampal SGL stem cell survival requires HOP function

**Figure 3:**
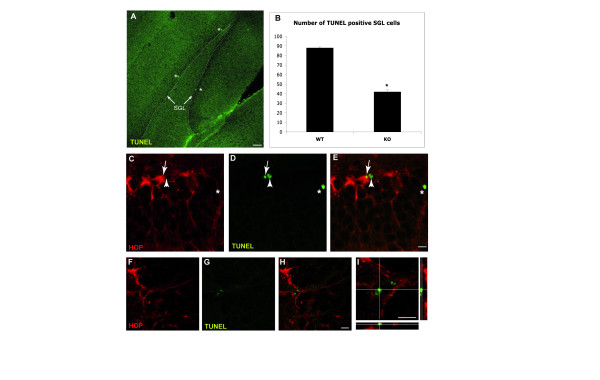
HOP regulates apoptosis of SGL astrocytic stem cells. (**a) **Apoptotic cells are detected in the SGL (arrows) with a TUNEL fluorescent assay. These cells are relatively rare (asterisks). Scale bar: 50 μm. (**b) **The number of TUNEL positive apoptotic cells in the SGL was counted across the entire DG (36 slices per mouse). A significant decrease of 48.2% is observed in knock out (KO) mice compared to wild type (WT) (n = 5, Mann-Whitney *U *test, *p = 0.001). (**c-h) **Double labeling for HOP and TUNEL shows that a majority of the TUNEL positive SGL cells are HOP immunoreactive (arrows). The arrowhead in (c-e) indicates a HOP negative TUNEL positive SGL cells (probably a D/type2 cell). The asterisk in (c-e) shows a TUNEL positive cell in the granule cell layer (probably a granular neuron). **(i) **Higher magnification of (h) with orthogonal projections along the z confocal stack at the x and y levels indicated by the intersecting white lines. Scale bars: 10 μm.

To evaluate a possible role of HOP in stem cell survival, we analyzed the number of TUNEL^+ ^SGL cells in wild-type and *HOP*^-/- ^mice (Figure 3a,b). We found that loss of *HOP *reduced the number of apoptotic SGL cells by more than two-fold (Figure 3b; n = 5, Mann-Whitney *U *test, *p *= 0.001). The amount of TUNEL^+ ^cells expressing *HOP *was 82% ± 4 SEM (223 cells of 3 animals), indicating their SGL astrocytic and thus stem cell nature (Figure 3c–i). TUNEL^+^/HOP^- ^cells are likely to be committed neuronal precursors (D/type 2 cells) or neurons, which are normally pruned [28]. Given that a large majority of apoptotic SGL cells in wild-type animals are HOP^+ ^and that this number is reduced in the absence of HOP, we conclude that HOP is a pro-apoptotic factor in SGL radial astrocytic stem cells in the hippocampus.

### HOP regulates granule neuron production in the hippocampus

**Figure 4:**
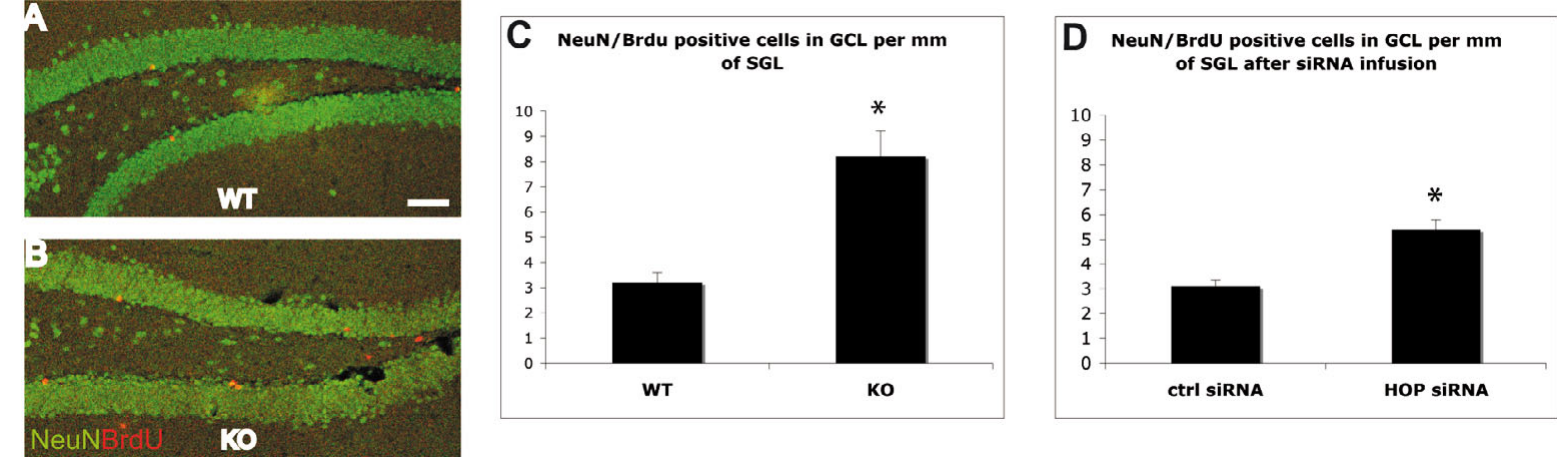
HOP influences neuronal production. **(a-c) **Wild-type (WT) and knock out (KO) mice were injected three times with BrdU (30 mg/kg) three weeks before perfusion and double staining was performed with NeuN and BrdU to visualize newly formed neurons in the granule cell layer. Double-labeled cells were counted under a confocal microscope (a, b) and the data are presented as the number of NeuN/BrdU positive cells per mm of SGL (c). The number of newly formed NeuN/BrdU neurons is significantly higher (2.5-fold) in knock out mice than in wild type (n = 6, Mann-Whitney *U *test, *p < 0.01). **(d) ***HOP *or control siRNA were infused above the DG for three days before three injections of BrdU. Three weeks later, the number of newly formed NeuN/BrdU neurons was significantly higher (1.7-fold) in the *HOP *siRNA infused mice (n = 5, Mann-Whitney *U *test, *p < 0.01). Scale bars: 50 μm.

An effect in stem cell survival should be reflected on the rate of production of new neurons. To assess this possibility, wild-type and *HOP*^-/- ^mice were pulsed with BrdU followed by a chase for three weeks before analyzing the number of new BrdU^+^/NeuN^+ ^DG neurons (Figure 4a,b), as NeuN selectively marks differentiated neurons but not progenitors. We found that the number of newly formed neurons was 2.5-fold higher in the absence of HOP compared to wild-type animals (Figure 4c; n = 6, Mann-Whitney *U *test, *p *< 0.01).

This result could be due to a function of HOP in SGL adult cells or to indirect effects, perhaps through an earlier effect of HOP. Indeed, only 50% of *HOP*^-/- ^animals survive to adulthood [1,2]. Thus, to resolve between these two possibilities, we tested the effects of an acute knock-down of HOP in the hippocampus of adult mice through RNA interference with cell-permeable siRNAs as described above. After three days of siRNA-Penetratin infusions, mice were pulsed with BrdU and kept for an additional three weeks to be able to assess newly formed neurons in the DG. This treatment led to a 1.7-fold increase in the number of newly formed NeuN^+^/BrdU^+ ^neurons in mice infused with *HOP *siRNA compared to mice infused with a control siRNA (Figure 4d; n = 5, Mann-Whitney *U *test, *p *< 0.01). The lower penetrance of the RNA interference phenotype (1.7-fold) versus that seen in knock out mice (2.5-fold) is consistent with the common incomplete effects of RNA interference compared with gene deletions. Notwithstanding this difference, the data support a direct role of HOP in the regulation of hippocampal stem cell apoptosis.

### Human glioblastomas display low levels of HOP expression

Faulty apoptotic control of stem cell lineages could underlie or favor the development of tumors. Gliomas are common adult human brain tumors that are thought to derive, at least in part, from stem cell lineages and harbor cancer stem cells [20-22,29,30]. We thus tested the possibility that loss of *HOP*, which decreases apoptosis in the normal mouse hippocampal stem cell niche, marks human gliomas. Here we have focused on grade IV astrocytomas (or GBMs), which are the most aggressive and invasive type of gliomas and display poor prognosis.

**Figure 5:**
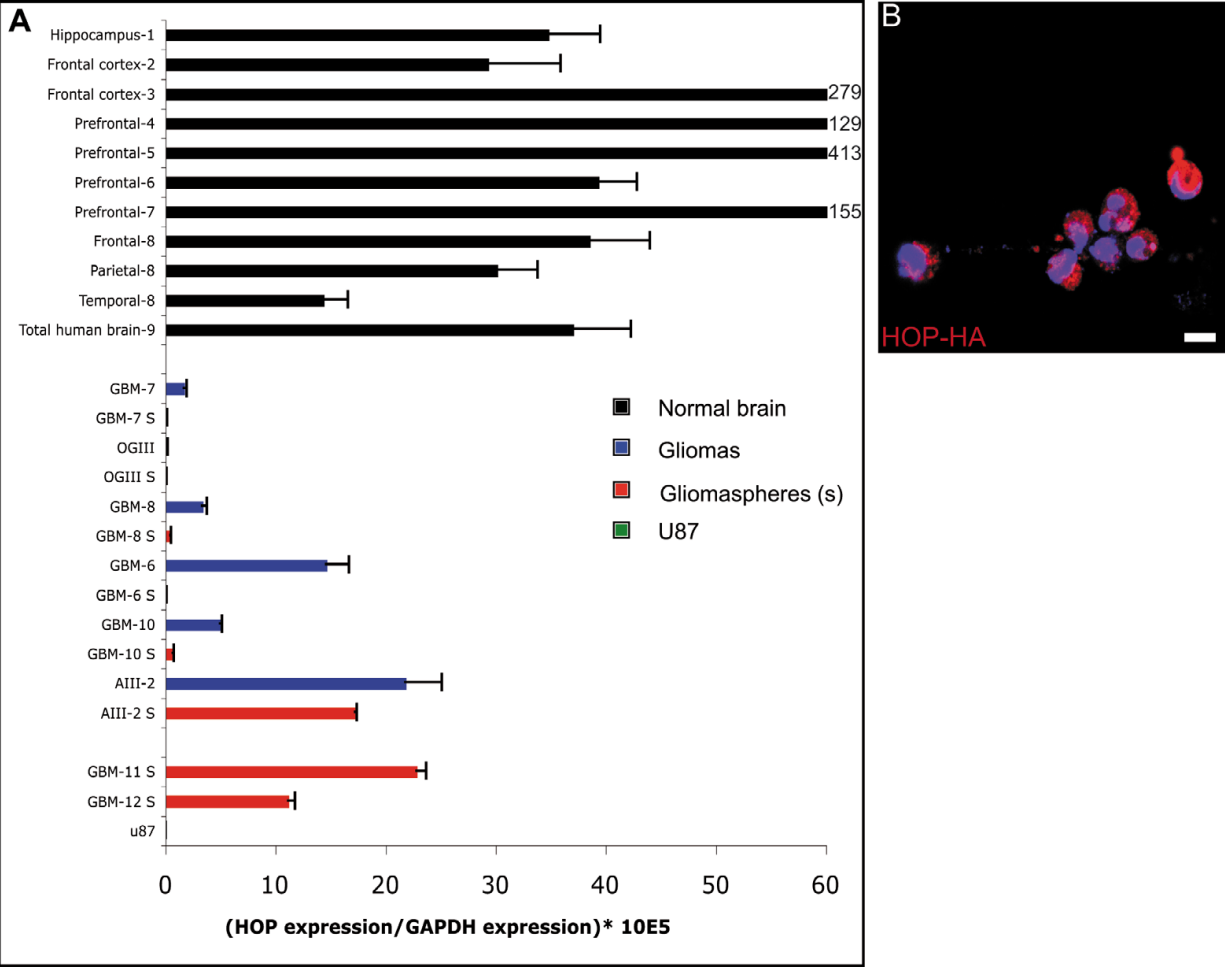
HOP in gliomas and gliomaspheres. **(a) **Expression levels of *HOP *in control cortical tissues (black bars), fresh glioma samples (blue bars), culture of gliomaspheres (red bars) or U87 cells (green bars). The data are expressed as the relative ratio of *HOP *expression over that of *GAPDH *times 10^5^. **(b) **Mouse HOP tagged with hemagglutinin (HOP-HA) and coupled to Penetratin was added to the medium of gliomaspheres from GBM-6 cells at 1 μM and the HA staining was developed. Cells are counterstained with Hoechst. Scale bar: 5 μm.

Analysis by quantitative RT-PCR of four independent glioblastomas [30] (GBM-6, -7, -8, -10) showed that *HOP *mRNA is down-regulated in tumor excisions compared to the normal cortex (Figure 5a). Nine normal brain samples were used: one sample of hippocampus from an adult donor, four samples of prefrontal cortex from different adult donors; three samples from the same child donor from three different regions of the cortex (frontal, parietal and temporal); and one commercial sample of total adult brain RNA. On average, expression of HOP was higher in normal samples than in glioma resections. Given the highly invasive nature of GBMs, surgically removed tumors are bound to contain a high proportion of normal brain cells. We thus measured HOP mRNA expression in glioma neurosphere cultures (gliomaspheres), derived from the same matched excision samples [30]. These cultures contain and are driven by self-renewing cancer stem cells and are tumorigenic, mimicking the original tumor after transplantation into the brain of nude mice [30,31]. *HOP *mRNA expression was very low in the four GBM stem cell cultures (Figure 5a). It was also extremely low in U87 GBM cells. *HOP *mRNA levels were higher in two other gliomasphere cultures (GBM-11 and -12), but we could unfortunately not compare these levels to the matched biopsies. Interestingly, the *HOP *expression level was also extremely low in one oligodendroglioma and corresponding gliomasphere culture. There is thus a positive trend correlating low levels of *HOP *and glioma stem cell lineages in a subset of gliomas.

### Increased HOP function decreases the survival, but not the proliferation, of a subset of human GBM stem cell lineages

To assess HOP function in GBM cells, we increased HOP function by introducing cell-permeable HOP. Purified full-length HOP protein, which itself is not cell permeable, was coupled with Penetratin peptide, thus rendering it cell permeable and functional in GBM cells after internalization (Figure 5b). As control, we used the homeodomain of Knotted (Kn-HD), a plant homeoprotein, which can naturally penetrate into cells [32]. Analysis of Ki67^+ ^cycling cells in GMB-6 stem cell cultures showed no difference in the percentage of proliferating cells after internalization of HOP-Penetratin versus Kn-HD (8.72% ± 0.75 SEM versus 8.53% ± 0.72 SEM). In contrast, HOP internalization triggered a 50% increase in apoptosis, as measured by the percentage of apoptotic cleaved-Caspase 3^+ ^cells compared to Kn-HD (13.9% ± 1.1 SEM versus 9.81% ± 0.87 SEM; *p *= 0.0001). Restored HOP function in human GBM-6 stem cell lineages is thus able to induce apoptosis without affecting proliferation. Similarly, HOP-Penetratin increased apoptosis in GBM-11 cells, which express high levels of *HOP*, compared with Kn-HD (10% ± 0.9 SEM versus 5.81% ± 0.46 SEM; *p *= 0.05), whereas no difference in their proliferation was measured by Ki67 staining (1.76% ± 0.05 SEM versus 1.7% ± 0.12 SEM). In contrast, still in comparison with Kn-HD, HOP-Penetratin did not modify apoptosis in GBM-8 cells (9.7 ± 1.31 SEM versus 7.63 ± 1.05 SEM; *p *= 0.24), which express low levels of HOP, or in GBM-12 cells (3.75% ± 1.4 SEM versus 3.29% ± 0.47 SEM), which express higher levels. This also confirmed the lack of non-specific effects of HOP-Penetratin in GBM cells. HOP is thus able to induce apoptotic cell death only in a subset of gliomaspheres with varying levels of endogenous HOP. Analyses of the growth rates of these cultures revealed that HOP was effective only in those that grew poorly: GBM-12 gliomasphere cultures had three times more cells than GBM-6 or GBM-11 cultures after six days under identical conditions and starting with the same number of cells (M Vukicekic and ARA, unpublished). The basis of this difference is not known.

### HOP regulates the self-renewal of apoptosis-sensitive glioma cancer stem cells

To test if HOP could affect stem cell self-renewal, we used GMB-6 cells that show a clear increase in apoptosis when treated with HOP-Penetratin. GMB-6 gliomaspheres were treated as above but in suspension to allow internalization of HOP-Penetratin or Kn-HD as control and to be able to plate one cell per well in 96-well plates. After two weeks of growth, gliomaspheres were counted in triplicates assays. It should be noted that clonal analyses of glioblastoma cells is stringent even in the presence of 50% conditioned media since these cells do not readily grow in isolation. HOP induced a ten-fold reduction in the number of clonogenic units (gliomaspheres) compared with Kn-HD (0.3% ± 0.32 SEM versus 3% ± 0 SEM; *p *= 0.01). HOP is thus able to induce apoptosis of glioma stem cells and decrease clonogenicity, used here as a measure of self-renewal.

## Discussion

We find that HOP is expressed in the adult hippocampal stem cell niche from E14.5 to adulthood. Expression is in all DG astrocytes and at high levels in SGL radial astrocytes. HOP protein is not detected in the adult SVZ, the second site of adult neurogenesis, but it is present in Bergmann glial cells in the cerebellum (data not shown), the only other radial astrocytes in the adult brain, confirming and extending previous mRNA localization data [33].

We describe here a new mode of regulation of adult hippocampal neurogenesis through modulation of adult progenitor death. A recent study has shown that 85% of the variance in neurogenesis between strains could be explained by different cell-survival rates [34]. However, attention has been focused on newly formed neurons in the olfactory bulb [8,9] or DG [10,11,28], but not on their progenitors. In contrast, developmental studies have established that apoptosis contributes to the number of neural stem cells and progenitors [12,13]. In the adult brain, apoptotic cells are present in the SVZ and SGL [14], and, in the SVZ, some of these apoptotic cells are astrocytes [35]. A role for apoptosis on the regulation of SVZ stem cells is also suggested by their enhanced number in mice deficient for the pro-apoptotic genes *Bax *and *Bak *[36]. Our work extends this concept to adult DG stem cells and, furthermore, identifies HOP as a positive regulator of apoptosis in these cells.

As fewer hippocampal progenitors die in mice lacking HOP, one could predict that loss of HOP may lead to a change of the total number of progenitors. However, the number of GFAP^+^/BrdU^+ ^cells (reflecting the number of progenitors) is the same in *HOP *null and wild-type siblings (Figure 2b). We have also counted the number of SGL cells expressing Sox2, a marker of actively dividing astrocytic DG progenitors [37], and found no difference in the number of Sox2^+ ^SGL cells between wild-type and *HOP *null mice (not shown). The reduction of hippocampal progenitor death in mice lacking HOP, therefore, does not lead to an increase in their number. A reduction in progenitor death not leading to an increase in their abundance is puzzling, although it could be explained by a difference in the choice between symmetric and asymmetric divisions in wild-type versus *HOP *null hippocampal stem cells. Indeed, GBM cells that increase apoptosis in response to enhanced HOP levels also decrease their self-renewal (as measured by their clonogenicity). Therefore, there may be a mechanism that senses overall cell numbers and that regulates self-renewal and apoptosis in normal and tumorigenic stem cell lineages.

HOP has been shown to interact with SRF in the heart [1,2] where it acts as an inhibitor of SRF transcriptional activity. Specific SRF invalidation leads to embryonic lethal cardiac defects [38-40]. These mice display an abnormally thin myocardium due to increased apoptosis without changes in proliferation. This result is consistent with ours, suggesting that HOP in radial DG progenitors may bind SRF and inhibit its activity We have started to investigate this possibility and find that HOP is co-immunoprecipitated with SRF from hippocampal extracts (Additional file 2). It is also known that SRF promotes cell survival by activating the expression of the anti-apoptotic gene *Bcl2 *in embryonic stem cell and early embryogenesis [41]. One could thus hypothesize that HOP acts in DG progenitors by inhibiting SRF activation of the *Bcl2 *gene. We tried to test this hypothesis by comparing Bcl2 expression in wild-type and knock out mice by quantitative RT-PCR but since the vast majority of hippocampal cells express Bcl2, a potential difference in a minority of progenitors could not be quantified.

The deregulation of apoptosis in hippocampal stem cells could contribute to dysfunction and/or disease. We report a common down-regulation of *HOP *in glioma excisions (which contain both tumor and normal surrounding tissues) compared to controls and even lower levels of *HOP *in gliomaspheres (which only contain tumor-derived cancer stem cell lineages). Experimentally, increased HOP expression enhanced apoptosis in a subset of gliomasphere cultures. Differences in responsiveness to HOP might depend, for instance, on the region of origin of the tumor or on the presence of co-factors necessary for HOP pro-apoptotic function. The apoptotic-inducing activity of HOP, albeit context-dependent, may be related to its normal function, as brain tumors are thought to derive from the transformation of stem cells and/or progenitors in adult neurogenic regions. Indeed, alterations in the cellular mechanisms that control adult neurogenesis might contribute to tumorigenesis [20-22]. This is consistent with different studies describing HOP as a possible tumor suppressor because of its down-regulation in choriocarcinoma [5], head and neck squamous cell carcinoma [4] and lung cancer [3]. HOP might thus participate in the regulation of stem cell behavior both in normal and in tumorigenic contexts.

## Conclusion

We describe here a new function for the homeoprotein HOP as a negative regulator of hippocampal stem cell survival. Our work provides the first evidence that apoptosis of stem cells can regulate adult neurogenesis. In addition, *HOP *expression is low in gliomas and its re-expression in gliomaspheres induces apoptosis and a reduction of glioma cancer stem cell self-renewal in a subset of cases. We suggest that HOP acts as a tumor suppressor in the hippocampal stem cell niche, where its loss could favor the inappropriate survival of stem cells and their descendants, increasing cell numbers and contributing thus to tumor growth.

## Materials and methods

### HOP cloning and antibody production

HOP cDNA was cloned from hippocampal mRNA and subcloned into pGEM vectors for riboprobe synthesis and pGEX vectors for protein production. The recombinant HOP protein was purified and used to produce a rabbit polyclonal antibody (Animal Pharm Services, Healdsburg, CA, USA).

### Histology

The mice used were either Swiss or the HOP knock out mice from the Epstein laboratory [1] analyzed along with control wild-type littermates.

For immunostaining, *in situ *hybridization and TUNEL staining, adult mice (two months old) were perfused (4% paraformaldehyde), their brains were removed, post-fixed overnight and sectioned with a vibratome (frontal sections, 50 μm thick).

### Immunostaining and *in situ *hybridization

Sections were blocked in phosphate-buffered saline, 10% fetal calf serum, 0.3% triton and incubated in primary antibodies in the same buffer for 36 h at 4°C under agitation. Antibodies were mouse anti-GFAP (1:1,000; Sigma-Aldrich, Lyon, France), rat anti-BrdU (1:1,000; Accurate Chemicals, Westbury, NY, USA), mouse anti-NeuN (1:1,000; Millipore, Saint Quentin en Yvelines, France, rabbit anti-SRF (1:500; Santa Cruz Biotechnology, Santa Cruz, CA, USA), rabbit anti-Sox2 (1:2,000; Millipore), guinea-pig anti-3PGDH (1:200; Frontier Science, Hokkaido, Japan), guinea-pig anti-Dcx (1:1,000; Millipore) Fluorescent secondary antibodies were FITC, rhodamine or Cy3 anti-species (1:1,000; Jackson Immunoresearch, Suffolk, UK). Immuno-labeled sections were observed and imaged with confocal Leica and Zeiss microscopes and a compound Axiophot microscope. *In situ *hybridizations were performed as described [42].

### TUNEL staining

Sections were dehydrated in successive baths of 50%, 70%, 90%, and 100% ethanol followed by rehydration along the inverse path. They were then incubated in the TUNEL (terminal deoxynucleotidyl transferase biotin-dUTP nick end labeling) staining mix of the Roche kit (Roche Diagnostics, Meylan, France) according to the manufacturer's instructions, thus leading to a FITC staining of apoptotic cells

### siRNA-Penetratin coupling

The sequence of the sense strand of *HOP *siRNA is: AAGGCTTGCCTTCGGAATGCA. It was produced by Qiagen (Courtaboeuf, France) with a thiol modification on the 5' extremity of the sense strand. Annealed siRNA duplex was resuspended in the buffer provided by the manufaturer and heated at 90°C for 1 minute followed by a 1 h incubation at 37°C. It was then pretreated with an equimolar quantity of TCEP for 5 minutes at room temperature. An equimolar amount of activated penetratin was added and the reaction was allowed for 2 h at 37°C. The efficacy of the coupling was assessed on 22% polyacrylamide gel with and without dithiothreitol. The control siRNA was the standard control siRNA from Qiagen called Negative.

### Brain infusions

Coupled siRNAs (20 μM) or growth factors (EGF + bFGF, 20 μg/ml) were diluted in 0.09% NaCl. Mice were infused continuously for three days using micro-osmotic pumps (1 μl/h, model 1003, Alzet, Cupertino, CA, USA) implanted streotaxically. The coordinates for intraventricular infusion of growth factors were: anterior/posterior, 0; lateral, 0.8 mm;depth, 2 mm (relative to bregma and surface of the brain). The coordinates for intraventricular infusion of siRNAs were: anterior/posterior, -0.82 mm; lateral, 1.5 mm; depth, 1.7 mm. The coordinates for intraventricular infusion of siRNA above the DG were: anterior/posterior, -2.46; lateral, 0.7 mm;depth, 2 mm.

### Quantitative analyses

At least five mice per condition were used. Statistical significance was determined using Mann-Whitney *U *test. After perfusion, the region of the brain containing the DG was cut frontally with a vibratome as four alternate series of nine 50 μm thick sections. For the proliferation assay, mice were injected intraperitoneally with BrdU (30 mg/kg) and perfused 2 h later. One of the four series of sections was immunohistochemically labeled with GFAP and BrdU. The sections were observed under the 20× objective of a Leica confocal microscope and all BrdU positive cells located in the SGL were zoomed upon (magnification of at least 5 times) and observed along the z-axis to assess their GFAP immunoreactivity. The DG length of each section was measured with NIH image and the total number of GFAP/BrdU positive cells of all sections was divided by this length to be expressed as GFAP/BrdU positive cells per DG length. For neuronal production, mice were injected intraperitoneally 3 times every 3 h with BrdU (30 mg/kg), perfused 3 weeks later and the quantification was performed as above after a NeuN/BrdU immunocytochemistry (ICC). For TUNEL staining, all 36 DG sections of each animal were processed and the total number of TUNEL positive SGL cells counted with the Leica SP2 confocal microscope.

To assess the reduction of immunoreactivity after siRNA infusions, the optical density of one series of sections per animal was measured with ImageJ as the mean gray value within the DG.

### Glioma tissues and stem cell cultures

Human glioma samples were obtained from the Hôpital Universitaire de Genève under approved protocols as in Clement *et al*. [30]. Glioma stem cell cultures were obtained by dissociating the fresh tumor sample from the operating room in 3 mg/ml Papain for 45–90 minutes followed by overnight culture in DMEM-F12, 20% BIT (Stem Cell Technologies, Grenoble, France), 1% penicillin-streptomycin. The cultures were then centrifuged, washed and cultured in full media (the same media as above plus 50% conditioned media from other gliomasphere cultures and 10 μg/ml each of recombinant bFGF and EGF). Manipulation of gliomaspheres was as in Clement *et al*. [30]. GBM U87 cells (ATCC, Molsheim, France) were grown as suggested by the supplier. Total human brain RNA was purchased from Yorkshire Biosciences Ltd (Heslington, York UK).

For internalization, gliomaspheres were cultured on poly-ornithine and poly-laminine coated wells and HOP coupled to Penetratin (Hop-Penetratin) or Knotted homeodomain were added to the medium at 1 μM (six wells per condition, repeated two times). After 48 h, cells were fixed with 4% paraformaldehyde and stained with rabbit anti-activated Caspase-3 (Cell Signaling, Beverly, MA, USA) or mouse anti-Ki-67 (Transduction Laboratory, Lexington, NY, USA). Stained cells were counted with a Zeiss Axiophot optical microscope.

For cloning assays, gliomaspheres were dissociated manually with a 200 μl tip. Dissociated cells were then treated with HOP-Penetratin or controls for 48 h before plating in full media in 96-well plates at a density of one cell per well. Fourteen days after plating, gliomasphere growth was scored visually under the microscope.

### Real time quantitative RT-PCR

Quantitative real time RT-PCR was performed using the iQ™ SYBR Green supermix (Biorad, Hercules, CA, USA) at 60°C, according to the manufacturer's instructions. Primers were 5' to 3': *HOP*-s, ACCACGCTGTGCCTCATC; *HOP*-a, TTGGTTAAGCGGAGGAGAGA; *GAPDH*-s, TGACATCAAGAAGGTGGTGAAGC; *GAPDH*-a, CCCTGTTGCTGTAGCCGTATTC.

## Competing interests

The authors declare that they have no competing interests.

## Authors' contributions

ADT participated in the staining and counting procedures, MZ performed all the glioma/gliomasphere experiments, JE provided the HOP knock out mice, ARA conceived and supervised the glioma/gliomasphere experiments and conceptually revised the manuscript, IC and AP designed the study and collaborated in the writing of the manuscript, IC carried out the surgery experiments, staining and counting. All authors read and approved the final manuscript.

## Supplementary Material

Additional file 1 Proliferation of 3PGDH immunoreactive SGL cells. **(a-c) **After a 2 h pulse of BrdU, mice were processed for 3PGDH/BrdU immunocytochemistry. Some 3PGDH immunoreactive cells incorporate BrdU. (c) Higher magnification of merged (a) and (b) with orthogonal projections. Scale bar: 10 μm. **(d) **After a 2 h pulse of BrdU, mice were processed for 3PGDH/BrdU ICC and the double-labeled SGL cells were counted under a confocal microscope. The data are presented as the number of 3PGDH/BrdU cells per mm of SGL. The number of 3PGDH/BrdU cells is unchanged in the *HOP *knock out (KO) mice compared to the wild type (WT). Click here for file

Additional file 2 HOP interacts with SRF in the hippocampus. Western blot with HOP antibody shows that HOP is present in hippocampal extracts (total) and efficiently co-immunoprecipitated with SRF (IP SRF). Click here for file
