# Supplementary figures and images for: Regulation of survival in adult hippocampal and glioblastoma stem cell lineages by the homeodomain-only protein HOP
Source: Neural Dev. 2008 May 28;3:13. doi: 10.1186/1749-8104-3-13 (PMC2416439; doi:10.1186/1749-8104-3-13)

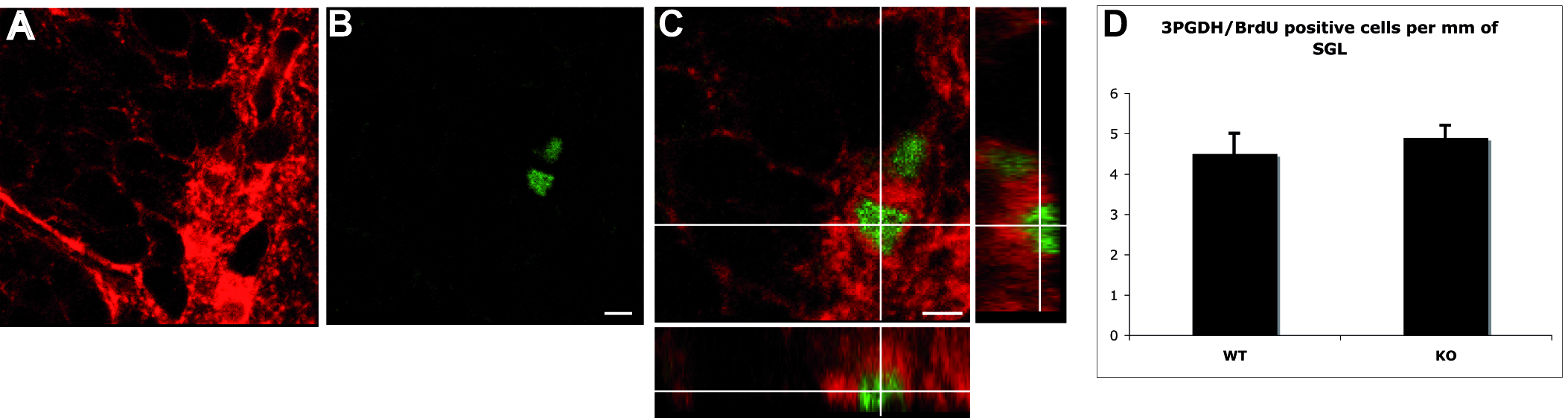

Supplement: Additional file 1 — Proliferation of 3PGDH immunoreactive SGL cells. (a-c) After a 2 h pulse of BrdU, mice were processed for 3PGDH/BrdU immunocytochemistry. Some 3PGDH immunoreactive cells incorporate BrdU. (c) Higher magnification of merged (a) and (b) with orthogonal projections. Scale bar: 10 μm. (d) After a 2 h pulse of BrdU, mice were processed for 3PGDH/BrdU ICC and the double-labeled SGL cells were counted under a confocal microscope. The data are presented as the number of 3PGDH/BrdU cells per mm of SGL. The number of 3PGDH/BrdU cells is unchanged in the HOP knock out (KO) mice compared to the wild type (WT). [file 1749-8104-3-13-S1.tiff]

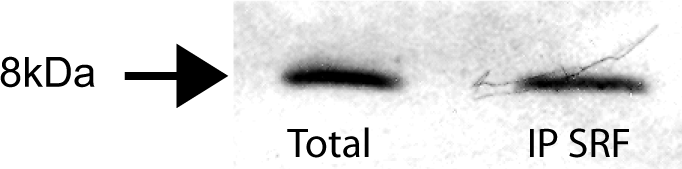

Supplement: Additional file 2 — HOP interacts with SRF in the hippocampus. Western blot with HOP antibody shows that HOP is present in hippocampal extracts (total) and efficiently co-immunoprecipitated with SRF (IP SRF). [file 1749-8104-3-13-S2.tiff]
